# Antibody and Cytokine Responses of Koalas (*Phascolarctos cinereus*) Vaccinated with Recombinant Chlamydial Major Outer Membrane Protein (MOMP) with Two Different Adjuvants

**DOI:** 10.1371/journal.pone.0156094

**Published:** 2016-05-24

**Authors:** Shahneaz Ali Khan, Marion Desclozeaux, Courtney Waugh, Jon Hanger, Jo Loader, Volker Gerdts, Andrew Potter, Adam Polkinghorne, Kenneth Beagley, Peter Timms

**Affiliations:** 1 Institute of Health and Biomedical Innovation, Queensland University of Technology, 60 Musk Ave, Kelvin Grove, QLD 4059, Australia; 2 Centre for Animal Health Innovation, Faculty of Science, Health, Education & Engineering, University of the Sunshine Coast, Locked Bag 4, Maroochydore DC, QLD 4558, Australia; 3 Endeavour Veterinary Ecology Pty Ltd, 1695 Pumicestone Road, Toorbul, QLD 4510, Australia; 4 Vaccine and Infectious Disease Organizations, International Vaccine Centre, University of Saskatchewan, 120 Veterinary Road, Saskatoon, Saskatchewan, Canada; 5 Faculty of Veterinary Medicine, Chittagong Veterinary and Animal Sciences University, Khulshi, Chittagong, 4202, Bangladesh; Midwestern University, UNITED STATES

## Abstract

Developing a vaccine against *Chlamydia* is key to combating widespread mortalities and morbidities associated with this infection in koalas (*Phascolarctos cinereus*). In previous studies, we have shown that two or three doses of a Recombinant Major Outer Membrane Protein (rMOMP) antigen-based vaccine, combined with immune stimulating complex (ISC) adjuvant, results in strong cellular and humoral immune responses in koalas. We have also separately evaluated a single dose vaccine, utilising a tri-adjuvant formula that comprises polyphosphazine based poly I: C and host defense peptides, with the same antigen. This formulation also produced strong cellular and humoral immune responses in captive koalas. In this current study, we directly compared the host immune responses of two sub-groups of wild *Chlamydia* negative koalas in one population vaccinated with the rMOMP protein antigen and adjuvanted with either the ISC or tri-adjuvant formula. Overall, both adjuvants produced strong *Chlamydia*-specific cellular (IFN-γ and IL-17A) responses in circulating PBMCs as well as MOMP-specific and functional, *in vitro* neutralising antibodies. While the immune responses were similar, there were adjuvant-specific immune differences between the two adjuvants, particularly in relation to the specificity of the MOMP epitope antibody responses.

## Introduction

Chlamydial infections are responsible for significant mortality and morbidity of mainland koalas (*Phascolarctos cinereus*) and are one major factor threatening the long term future of this iconic species [1–4]. The main species of *Chlamydia* that infects koalas is *C*. *pecorum*, and virtually all koala populations are infected, with rates ranging from 10% to as high as 90% in some regions [1]. Despite the significant advances in chlamydial research, a prophylactic vaccine to stabilize the population decline caused by chlamydial infections [5] has yet to be fully developed.

*Chlamydia* is an intracellular bacterium with a unique biphasic developmental cycle, consisting of two developmental forms, the non-dividing, but infectious, elementary bodies (EBs) and the replicative, but non-infectious reticulate bodies (RBs) [6]. It is usually accepted that a host requires the development of a balanced Th1 and Th2 protective immune response to adequately control chlamydial infections [7]. Several small animal studies have confirmed the protective role of IFN-γ secreting CD4+T cells in chlamydial infection [8]. Recently, there is also re-emerging evidence supporting the prominent role of B cells to elicit protective anti-*Chlamydia* antibodies [9]. The primary role of the neutralizing antibodies is to reduce the initial infectious burden and further prevent secondary bacterial infections [10]. Once the bacterium parasitises the host’s cells, the cell mediated immune response pathway contributes significantly to protective immunity through IFN-γ secretion [11]. Whilst IL-17A is a strong recruiter of neutrophils which secrete antimicrobial peptides and promote a Th1 immune response against intracellular pathogens [12], other animal studies suggest that IL-17 plays a role in both immune pathology and protection [13].

The chlamydial major outer membrane protein (MOMP) is the leading vaccine candidate in chlamydial vaccine research, and our group has been developing a prototype vaccine utilizing recombinant chlamydial MOMP (rMOMP) as a vaccine antigen for koalas. Although the choice of immunogenic antigen is of prime importance, selecting the right adjuvant to appropriately trigger the immune response is also essential. In this context, we have used two different adjuvant formulations with differing properties, combined with rMOMP, to vaccinate groups of koalas: ISC (immune stimulating complex) adjuvant [14–17] or Tri-adjuvant which is a mixture of the three components (Polyphosphazine, poly I: C and host defense peptides) [18].

In our previous koala vaccine trials, the ISC adjuvant was able to induce strong cellular and humoral immune responses [14–17]. However, the ISC adjuvant requires two or three injections to promote a significant immune response. This is logistically problematic for wild koalas, which would need to be tracked and re-captured, or kept in captivity for extended periods of time, increasing the cost of the process as well as the stress experienced by the animal itself. A trivalent adjuvant (Tri-Adj) containing polyphosphazine, poly I: C and host defense peptides, has been developed to be effective with just a single dose [18]. In other species, this adjuvant promoted a Th1 and Th2 balanced immune responses following a single injection [19–23]. In a small preliminary trial in captive koalas (n = 6), we have shown that this adjuvant was safe to use and elicited promising immune responses [18].

In the current study, we evaluated, in detail, both the cellular and humoral immune responses of wild koalas vaccinated with rMOMP, combined either with (a) the single-dose Tri-Adj or (b) three doses of ISC. Firstly, we evaluated the cellular response for each adjuvant by measuring cytokine gene expression elicited by the peripheral blood mononuclear cells (PBMCs) at defined post-vaccination time points. Secondly, we measured the neutralising antibodies produced by vaccination and mapped the corresponding MOMP epitopes recognized for both cohorts.

## Materials and Methods

### Koalas

The koalas used in our study were sourced from a wild population of around 400 animals located in South East Queensland. Prior to vaccination, all animals were examined and those animals that (i) had no clinical evidence of chlamydiosis; and (ii) were negative at conjunctival and genital sites following *Chlamydia pecorum*-species-specific qPCR screening [24] were selected and all animals were breeding age (>1 year) of either sex, as assessed during the initial capture event by qualified wildlife veterinarians. Two sub-sets of these animals have been vaccinated with an anti-*C*.*pecorum* vaccine and we analysed a further sub-set of these vaccinated animals in the current study. The first group of 10 koalas (Cindy, Greg, Cherry, Maxwell, Kylie, Paige, Janke, Squeek, Linky and Kelly) (Group A) were vaccinated with chlamydial rMOMP protein (see below for details) mixed with the Tri-Adj. A second group of 5 koalas (Robyn, Pepper, Maya, Hunky Harry and Winnic) (Group B) were vaccinated with rMOMP protein mixed with ISC [17]. At the end of the trial, all koalas were successfully returned to their habitat in accordance with regulatory approvals. None of the animals in our sub-study groups were diseased or required treatment or euthanasia during the study period. Animals were captured at a minimum of every 6 months and viewed from the ground weekly. All work was conducted under permission from Queensland University of Technology’s Animal Ethics Committee (AEC; Permit # 1200000122), the University of the Sunshine Coast AEC (ANA1380) and Scientific Purposes Permit (WISP11532912).

### Vaccines

Both vaccines consisted of *C*. *pecorum* rMOMP combined with either adjuvant (Tri-Adj or ISC) and were implementing through subcutaneous route. We combined three rMOMP proteins (A, F and G types) for the vaccine, as described previously [14, 17, 18]. Koala-specific *C*. *pecorum* MOMP proteins were expressed and purified as per Kollipara et al. [14]. The purified products were used for vaccination and ELISA assays. After vaccination, the animals were released back into the wild and tracked with a wildlife telemetry system (K-Tracker, LX Solutions Pty Ltd). The ISC vaccinated koalas were re-captured at 1 monthly interval to receive the 2^nd^ and 3^rd^ dose of the vaccine and a veterinary health examination.

### Samples

Aluminium shafted cotton-tipped swabs (Copan, Interpath Services, Melbourne) were used to collect samples from the conjunctiva of the left and right eye, as well as the urogenital sinus (prostatic urethra in males), as previously described [18]. These swabs were used for measuring the *C*. *pecorum* infection load using a *C*. *pecorum*-species-specific qPCR targeting the 16S rRNA gene [24]. Blood samples were obtained from the cephalic vein into EDTA-containing tubes and stored at 4°C for processing within 24 h of collection, to obtain PBMCs. After centrifugation at 1000 rpm for 5 mins, plasma was separated and used for ELISAs and *C*. *pecorum in vitro* neutralisation assays. The samples were collected at 0 (pre-vaccinated), 2 and 6 months post vaccination.

### Cytokine assays

The blood samples were centrifuged within 4–8 h of collection to separate the plasma. The PBMC were isolated by centrifugation on Ficoll-paque gradients (GE Healthcare, Rydalmere, Australia) washed and suspended in 1ml RPMI 1640 T cell media supplemented with 5% foetal calf serum, antibiotics and β-mercaptoethanol (0.001M) (Sigma) at a concentration of 2x10^6^ cells/ml. A 500 μl aliquot of cell suspension was used as the pre-stimulation sample. The remaining cells were then stimulated with either mitogens (Ionomycin and PMA combination)[25] or UV-inactivated *C*. *pecorum* EBs. After stimulation and incubation at 37°C with 5% CO_2_, the cells were collected at 12 and 24 h post-stimulation time points. RNA extraction and cDNA synthesis were completed for all these pre- and post-stimulation samples according to our previously published protocol (21). The end products were utilized in qPCR assays to determine the mRNA expression level as fold change for interferon gamma (IFN-γ), interleukin 17A (IL-17A), interleukin 10 (IL-10), tumour necrosis factor alpha (TNF-α) and glyceraldehyde 3-phosphate dehydrogenase (GAPDH) [25–27]. GAPDH was used as reference to normalise IFN-γ, IL-17A, IL-10 and TNF-α using the 2^-ΔΔCT^ method (ΔΔCT = (Ct of target gene—Ct of GAPDH) at 12 or 24 h time point–(Ct of target gene–Ct of GAPDH) at 0 time point [28].

### *C*. *pecorum* specific ELISA

Enzyme-linked immunosorbent assays were performed using purified rMOMP as per Kollipara et al. [14] and Khan et al.[18] on the plasma samples collected at 0, 2 and 6 month time points post-vaccination.

### *C*. *pecorum* MOMP peptide ELISA

We initially screened the plasma to identify the reacting epitopes for individual animals, using the methods described previously [9]. Then we measured the individual peptide concentrations as determined using our previously described ELISA methods [18]. Instead of using the whole rMOMP protein, we used selected peptides for coating the ELISA plates at a concentration of 2μg/well in PBST. Post-incubation, the wells were washed 3x with PBST and the plasma sample was serially diluted two fold at 1:200 dilution initially, and incubated at 4°C overnight. Plates were then washed 3x in PBST and a sheep anti-koala IgG (1:8000 in PBST;[16]) was added. At this point, plates were incubated for a further one hour at room temperature. After a further three washes (PBS-T), HRP-conjugated rabbit anti-sheep IgG (1:1000, Southern Biotech ⁄ Millipore, North Ryde, Australia) was added to wells and incubated at room temperature for 1 hr. Post incubation, plates were washed 4x with PBS and 50 μl ABTS [2, 2′-azino-bis (3-ethylbenzothiazoline-6-sulphonic acid), Southern Biotech, Alabama, USA] solution was added and incubated for 10 mins to observe the greenish color development. The reaction was stopped with 1M sulphuric acid following color observation. The optical density was measured at 405 nm wavelength and the data was transformed into excel sheet for later analysis.

### Koala-specific *C*. *pecorum* neutralising antibodies

We conducted *in vitro* neutralisation assays using the methodology of Kollipara et al. [14] either on whole plasma or on plasma collected at 0, 2 and 6 month time points which had been pre-adsorbed with one or more individual peptides [14]. All plasma samples were diluted at 1:10 prior to assay. The background neutralisation was determined by using koala plasma that was *Chlamydia* negative. Percentage neutralization was then determined by subtracting this background from each individual to determine the final neutralisation. The results were expressed as fold change neutralisation.

### *C*. *pecorum* MOMP peptide mapping

Biotinylated Pepscan ELISA was performed as previously described [9] to identify the specific rMOMP epitopes produced by each vaccine in animals receiving either the ISC or Tri-Adj adjuvants. Briefly, we designed 88 peptides with 15-mer peptides that spanned the full length of koala *C*. *pecorum* MOMP F protein and used these individually in ELISA assays (or grouped) as described above. The background for each plasma sample was calculated from the mean plus twice the standard deviation of the negative wells (no plasma added). We scored samples with an absorbance value greater than 0.5 as a positive response. In subsequent experiments, we utilised only the positive peptides to coat the streptavidin plate at a concentration of 2μg/well and performed the standard ELISA as described.

### *C*. *pecorum* MOMP-peptide specific neutralising antibodies

We performed three types of neutralising assays by using the (a) whole plasma at 1/10 dilution for either Tri-Adj or ISC cohort, (b) whole plasma at post-adsorption against either peptide 58 and 77 for tri-adjuvant or at post-adsorption against epitope 4 for ISC cohort and finally (c) whole plasma at post-adsorption against either epitopes 4, 28, 41, 42, 58, 59 and 77 for Tri-Adj cohort or 4, 28, 41, 42 for ISC cohort. We utilised the previously described novel protocol for peptide adsorption [9].

### Statistical analysis

Statistical analyses were performed using Graph-Pad Prism version 6 (Graph Pad Software, La Jolla, CA, USA) and the P value for significance was set at ≤ 0.05. Data between cohorts was analysed using one–way ANOVA Kruskal-Wallis (non-parametric) tests.

## Results

### There was a non-significant trend towards stronger IFN-γ and IL-17A responses in animals immunised with the Tri-Adj compared to ISC immunised animals

**Fig 1:**
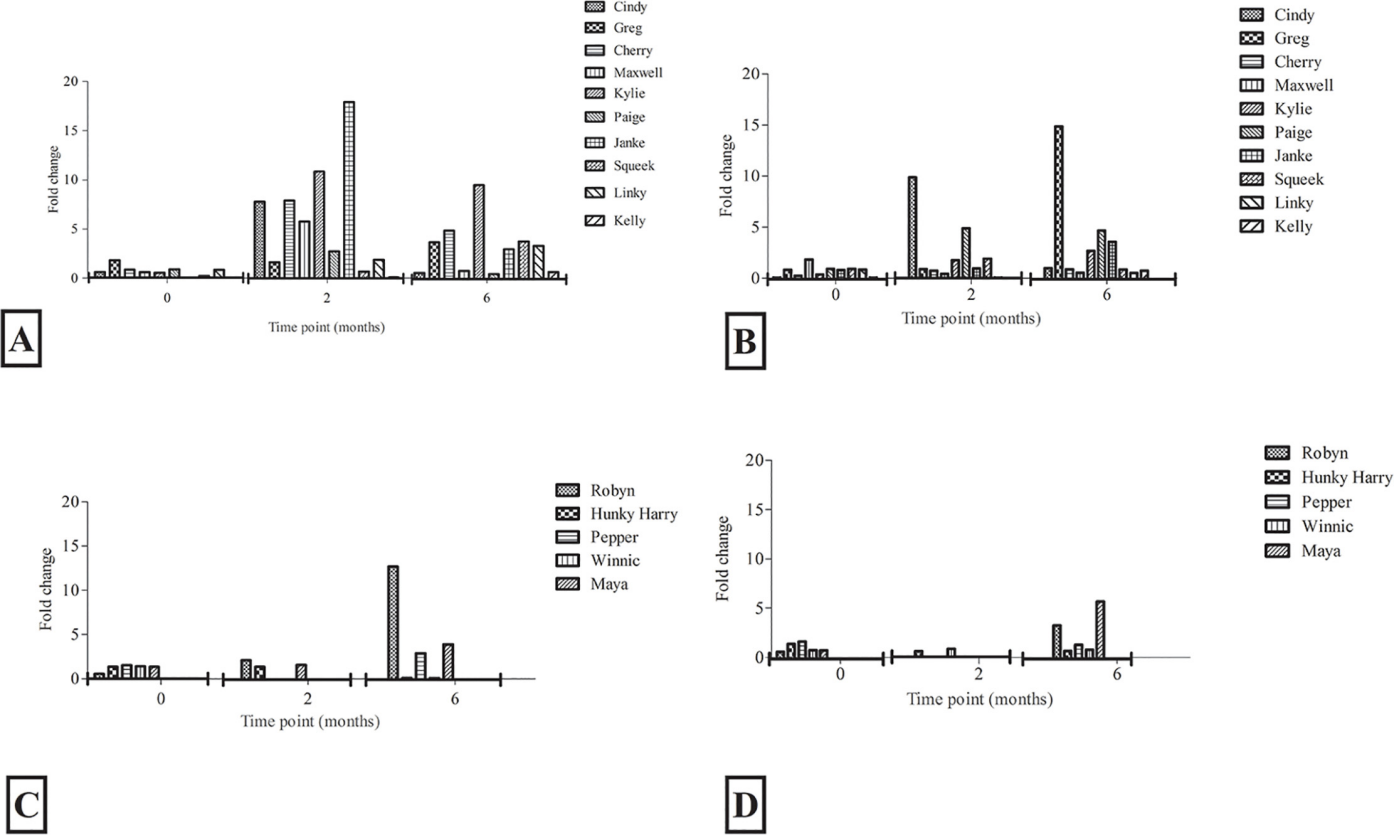
IFNγ (A, C) and IL17A (B, D) gene expression in koala PBMCs stimulated with UV inactivated *C*. *pecorum* at 0, 2 and 6 months post vaccination. The Tri-Adj (A, B) and ISC (C, D) cohort’s response are presented together (Fig 1 A-D). Results are expressed as fold increase compared to internal control gene GAPDH.

**Fig 2:**
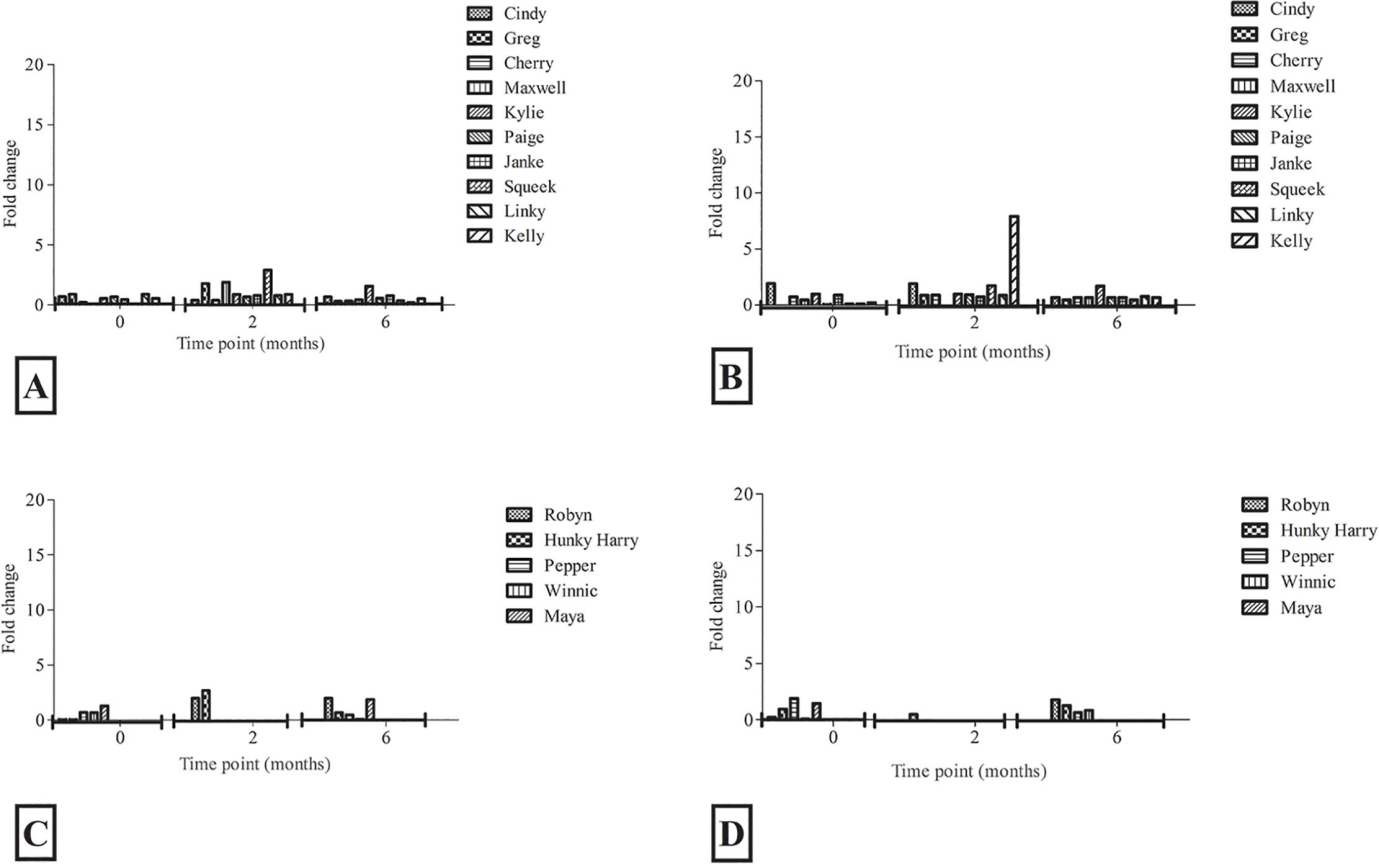
IL10 (A, C) and TNFα (B, D) gene expression in koala PBMCs stimulated with UV inactivated *C*. *pecorum* at 0, 2 and 6 months post vaccination. The Tri-Adj (A, B) and ISC (C, D) cohorts are presented together (Fig 2 A-D). Results are expressed as fold increase compared to internal control gene GAPDH.

To evaluate differences in the immune response of koalas vaccinated with a *C*. *pecorum* rMOMP-vaccine adjuvanted with either Tri-Adj or ISC, we vaccinated a cohort of koalas that were clinically healthy at the time of vaccination and were *Chlamydia* PCR negative at both urogenital and ocular sites (data not shown). Immune profiling of these vaccinated animals revealed that 60% of the animals in both groups produced IFN-γ at 2 or 6 months post vaccination in response to stimulation of PBMCs with UV-inactivated EBs (elementary bodies) (6 out of 10 for Tri-Adj and 3 out of 5 for ISC adjuvant). For those animals whose PBMCS expressed IFN- γ in response to stimulation, the level of IFN-γ expression varied from 2.73 to 17.89-fold for Tri-Adj and from 2.08 to 12.67-fold for ISC (Fig 1). We also observed differences among the responders between the 2 month and 6 month time points. For the Tri-Adj responders the highest expression was observed at 2 months, whereas, for ISC responders the highest IFN-γ responses were at the 6-month time point. Overall, the IL-17A responses were lower than IFN-γ, and only 40% of animals (4/10 Tri-Adj; 2/5 ISC) produced IL-17A responses to stimulation above 1.0 fold. We did not observe any measurable expression for the anti-inflammatory cytokine, IL-10 and TNF-α following stimulation of collected PBMCS from animals in either cohort (Fig 2A–2D).

### The kinetics of the total antibody (IgG) titres was similar in both cohorts, though there was an increased trend towards higher plasma IgG titres in ISC cohorts

**Fig 3:**
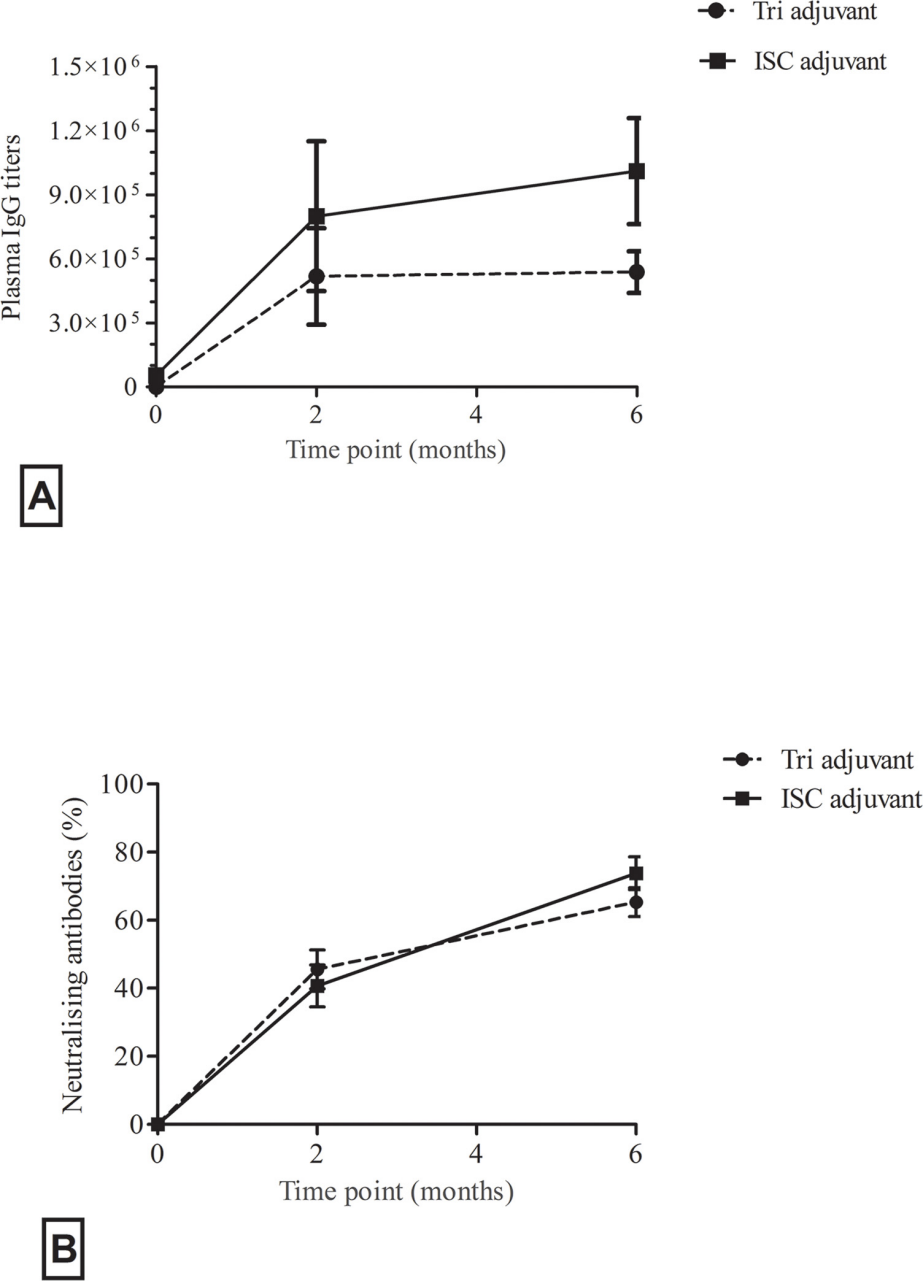
rMOMP specific IgG in plasma of vaccinated koalas was assayed by ELISA at 0, 2 and 6 months post vaccination. IgG levels are expressed as end-point titers (EPT) and represent the mean ± SD of 10 and 5 koalas in the Tri-Adj and ISC cohort respectively (Fig 3A). Vaccine induced *C*. *pecorum* percent neutralisation in plasma is presented (Fig 3B) compared to pre-immunisation samples. All samples were assayed at 1:10 dilution and *C*. *pecorum* EBs (50,000 IFU) were added to samples. The results are expressed as the percentage neutralisation of post-immunized samples compared to that of the pre-immunized and non-infected samples. Results are expressed as the mean ± SD of 10 and 5 koalas in the Tri-adj and ISC cohort respectively. There was no significant difference between the two cohorts.

**Fig 4:**
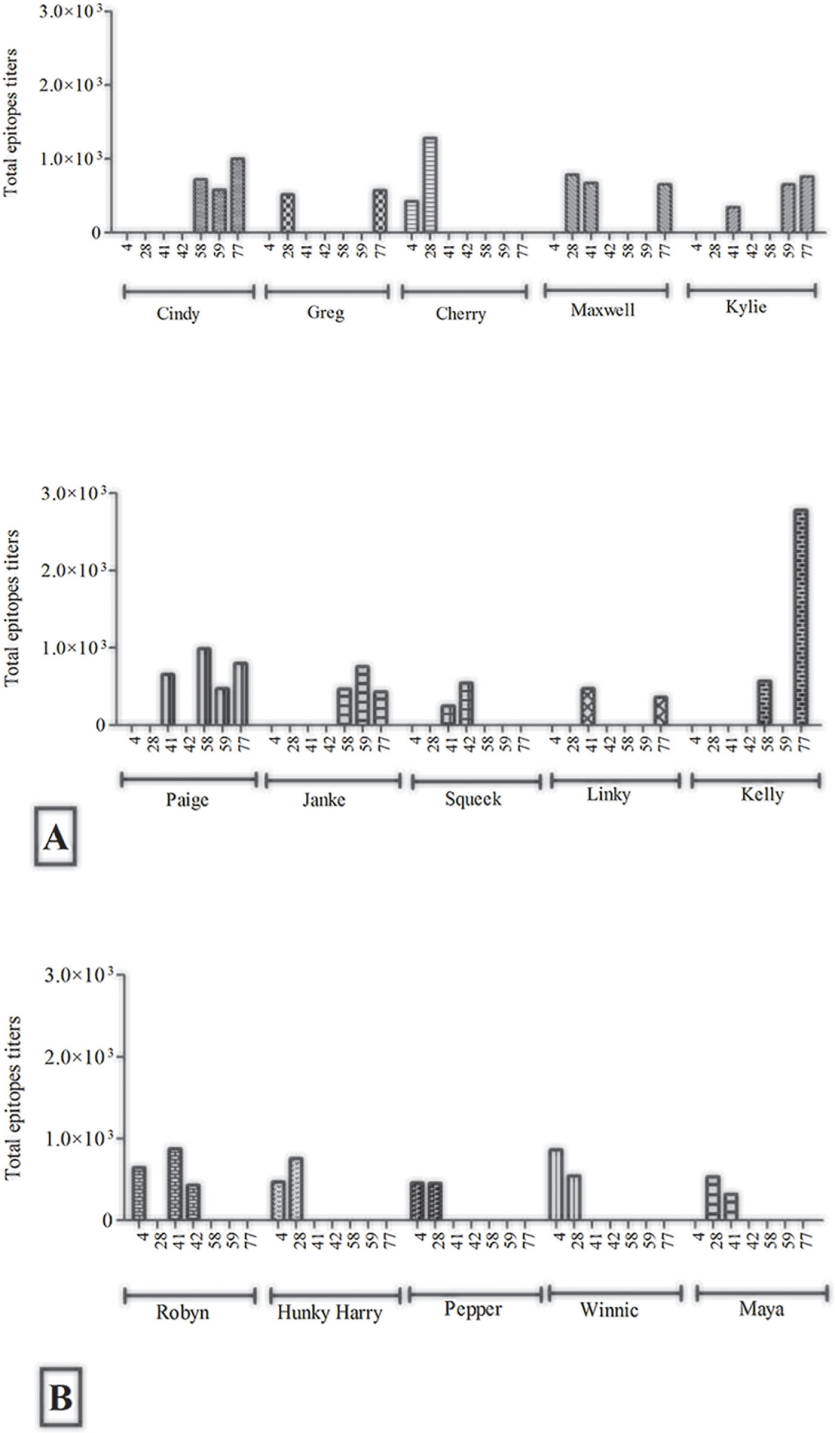
ELISA titers against individual peptide. Panel A (10 animals) for the Tri-adjuvanted vaccinated animals and panel B (5 animals) for the ISC cohorts. We did not consider epitope 86 as this epitope was detected in naturally infected koalas in our previous study.

Both vaccine formulations elicited strong anti-MOMP antibody levels following vaccination. The Tri-Adj cohort produced titres of around 5x10^5^ at 2 months post-vaccination, which persisted up to 6 months. The ISC cohort produced a similar average titre at 2 months (7x10^5^) which increased (to 9x10^5^) by the 6 months time point (*p* value 0.302) (Fig 3A). We also measured the antibody responses to individual peptides (selected ones) by ELISA (Fig 4A; 4B). The titres for the individual epitopes varied from 0.3x10^3^ to 2.8x10^3^ EPT. Interestingly, there was very little difference in titres for the individual epitopes, except for epitope 77 in a single koala (Kelly) (Fig 4A).

### Similar *C*. *pecorum* specific neutralising antibody potential was produced by both adjuvants

To compare the function of antibody responses induced by either vaccine formulation, *in vitro* neutralisation assays were performed with plasma from the Tri-Adj and ISC cohorts at 2 and 6 months post-vaccination. All samples were diluted 1:10 prior to testing their neutralising ability on *C*. *pecorum* infected cell culture mono-layers. Both adjuvant cohorts produced almost identical *in vitro* neutralisation levels, with both groups having increased neutralisation levels at 6 months compared to 2 months post vaccination (Fig 3B).

### Epitope mapping identified two distinct anti- *C*. *pecorum* MOMP peptide antibody profiles for the two adjuvant groups

We used the Pepscan approach [9] to examine the epitope specificity of the plasma antibody response to vaccination in our Tri-Adj versus ISC adjuvant groups. In total, four *C*. *pecorum* MOMP peptides (4, 28, 41, and 42) were recognized in our *C*. *pecorum* peptide ELISA from animals in each cohort with an additional three peptides recognised by koalas receiving the Tri-Adj formulation only (58, 59 and 77). There was variability in the responses to these individual peptides with peptide 4 recognised by 80% (4 out of 5) of the ISC cohort but only by 10% (1 out of 10) of the tri-adjuvant cohort. For the other epitopes, none were recognised by 100% of the animals in any cohort, although the most-broad recognition by the animals was with epitopes 77 (8/10 of tri-adjuvant animals), 58/59 (5/10 tri-adjuvant animals), 41/42 (5/10 tri-adjuvant cohort) and 28 (4/5 ISC animals) (S1 Fig; Table 1).

**Table 1:** Epitope mapping of antibodies in plasma samples at 6 months post vaccination as determined by Pepscan assay. CD: MOMP Conserved domain; VD F1: Variable domain 1 for MOMP F; VD F2: Variable domain 2 for MOMP F; VD F3: Variable domain 3 for MOMP F; VD F4: Variable domain 4 for MOMP F; VD A: Variable domain 1, 2, 3, 4 for MOMP A; VD G: Variable domain 1, 2, 3, 4 for MOMP G; VD H: Variable domain 1, 2, 3, 4 for MOMP H.

**Tri-Adjuvant cohort**	**CD**	**VD F1**	**CD**	**VD F2**	**CD**	**VD F3**	**CD**	**VD F4**	**CD**	**VD A**	**VD G**	**VD H**	**CD**
Cindy									58, 59		77		
Greg			28								77		
Cherry	4		28										
Maxwell			28				41				77		
Kylie							41		59		77		
Paige							41		58, 59		77		
Janke									58, 59		77		
Squeek							41, 42						
Linky							41				77		
Kelly									58		77		
**ISC cohort**	**CD**	**VD F1**	**CD**	**VD F2**	**CD**	**VD F3**	**CD**	**VD F4**	**CD**	**VD A**	**VD G**	**VD H**	**CD**
Robyn	4						41, 42						
Hunky Harry	4		28										86
Pepper	4		28										
Winnic			28										
Maya	4		28				41						

### The vaccine induced anti-epitope antibodies had neutralising ability, either individually or in synergy with other epitopes

**Fig 5:**
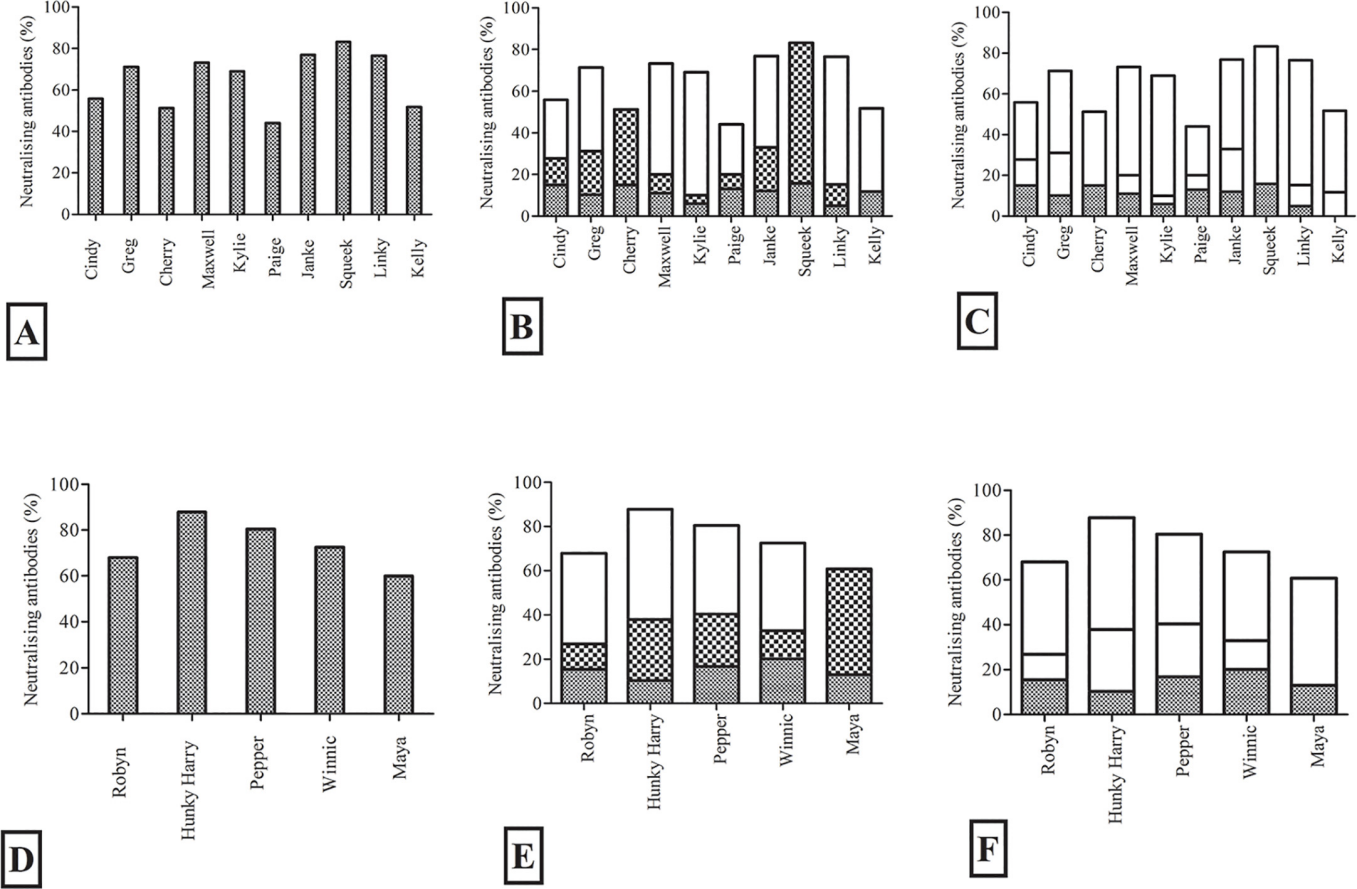
*In vitro* neutralisation levels of plasma from vaccinated koalas (A) whole plasma at 1/10 dilution for Tri-Adj cohort, (B) whole plasma after absorption against peptides 58 and 77, or (C) whole plasma after adsorption against peptides, 4, 28, 41, 42, 58, 59, and 77. Separately, the *in vitro* neutralisation of plasma from ISC cohort (D) whole plasma at 1/10 dilution, (E) whole plasma after adsorption against peptide, 4, (F) whole plasma after adsorption against peptides, 4, 28, 41 and 42. The neutralising antibodies were presented against individual animals and all samples were diluted at 1:10 dilution and *C*. *pecorum* EBs (50,000 IFU) were added to samples. The results are expressed as the percentage in neutralisation of immune samples compared to that of the pre-immune and non-infected samples. The reductions of neutralising antibodies are presented as empty spaces in the bar. (Panel B: Empty space represents the neutralising anti-epitopes 58 and 77; Panel C: Empty space represents the neutralising anti-epitopes 4, 28, 41, 42, 58, 59 and 77; Panel E: Empty space represents the neutralising anti-epitopes 4; Panel F: Empty space represents the neutralising anti-epitopes 4, 28, 41 and 42).

We examined the contribution of antibodies against individual epitopes or groups of epitopes, to the observed *in vitro* neutralisation effect. We compared (a) whole plasma versus (b) plasma pre-adsorbed against the most recognized peptides 58, 77 for Tri-Adj, and peptide 4 for ISC versus (c) plasma pre-absorbed against epitopes 4, 28, 41, 42, 58, 59, 77 for Tri-Adj and 4, 28, 41, 42 for ISC. We evaluated the neutralising ability of each of these pre- and post-absorption samples and compared the relative reduction of neutralisation ability in each case (Fig 5B and 5E). We found that most (if not all) of the individual anti-epitope antibodies contributed to *in vitro* neutralisation. In the case of the Tri-Adj vaccinated animals, anti-58 and anti-77 epitope antibodies made a major contribution to the *in vitro* neutralisation effect (white bars in Fig 5B). The effect of these antibodies was confirmed with animal “Cherry” (did not produce any anti-58 antibodies) and animal “Squeek” (did not produce any anti-77 antibodies) as the *in vitro* neutralisation level for these animals was not reduced following absorption against 58 or 77 peptides. We also observed significant *in vitro* neutralisation by antibodies against peptides 4, 41/42 and 28, with anti-peptide 4 antibodies (especially in the ISC cohort), having a major effect (Fig 5E).

## Discussion

Our previous work suggested that the koala’s immune system is able to mount both effective cellular and humoral immune responses against a rMOMP vaccine, when administered in combination with two different adjuvant systems [14–18]. While both adjuvant vaccines look promising, one requires two or three doses (ISC) while the other is a single administration vaccine (Tri-Adj). We therefore decided to directly compare the immune responses of the two vaccine formulations using the same rMOMP antigens to vaccinate koalas from the same wild population.

Studies with the mouse model of *C*.*muridarum* show that an IFN-γ response is required for adequate protection against chlamydial infections. While there is no direct evidence yet for protection against *C*. *pecorum* infections in koalas, vaccine development should aim for a strong IFN-γ response. We found that both vaccine formulations induced good IFN-γ responses in 60% of animals that lasted for up to 6 months. No significant difference could be seen in the specific IFN-γ response induced by the single dose Tri-Adj formulation or the ISC formulation. IFN-γ activity is the hallmark of the Th1 immune response against chlamydial infection and IFN-γ gene knockout mice are indeed unable to resolve the infection [29]. Despite the promising IFN-γ response in some animals (60%), not all koalas produced a detectable IFN-γ response. The animals in this trial are outbred animals and this highlights key Major Histocompatibility Complex (MHC) considerations for future vaccine development. Genetic differentiation and structure analysis has revealed that the koala’s MHC-II gene is more diverse in koalas in the northern states of Queensland and New South Wales, compared to the southern states of Victoria [30, 31]. The higher MHC-II diversity could be the potential cause of the variable immune response within this group of koalas.

In addition to IFN-γ, IL-17A has been suggested as an important cytokine for chlamydial infection, both for protection, but potentially also in disease pathology [13, 32]. We observed *Chlamydia*-specific IL-17A responses for 40% of koalas with both vaccine formulations. Whilst, recent studies in koalas [27] and women [33] reported that strong expression of IL-17A has been associated with clinical chlamydiosis and chlamydial cervicitis, IL-17-/- mice showed less pathological lesions compared to BALB/c [13]. Moreover, an elevated IL-17A response has been observed in clinical chlamydial infection in mouse model [34]. Though the mechanism of IL-17A in pathogenesis is unclear, this study confirms both vaccines can induce expression of this cytokine.

While we did not observe any measurable anti-inflammatory cytokines response in either group, still their role in chlamydial immunity and pathogenesis is controversial. In general, IL-10 suppresses the secretion of various pro-inflammatory cytokines involved in chlamydial pathogenesis [35]. Furthermore, in the mouse model, the IL-10 dominated response has been attributed with susceptibility to chronic infection [36]. A similar observation has been seen in trachoma infected populations [37]. The higher expression of the IL-10 gene promoter has been associated with increased chlamydial infection and disease severity [38]. Similarly, a higher level of IL-10 has been linked to *C*. *trachomatis* infertility [39, 40] and tubal damage in women [41]. However, koalas with clinical chlamydiosis, expressed IL-10 in variable levels, with some animals showing higher levels of expression similar to IFN-γ [25, 27]. In a similar fashion, the role played by TNF-α in chlamydial infection has provided disparate results. However, TNF-α has been linked to an initial clearance of primary infection but challenge infection elicited immune-pathology in the mouse [42] and guinea pig model [43]. In the mouse model studies showed TNF-α produced by CD8+ T cells, promote inflammation in the oviduct following *C*. *muridarum* infection [44] but CD4+ T cells producing IFN-γ and TNFα are generally immune-protective. In contrast, reduced chlamydial shedding following challenge infection in vaccinated mice, has been attributed to the co-expression of TNF-α and IFN-γ [45].

While cytokines are considered to be the major immune mechanism for protection against chlamydial infections, antibodies continue to be considered just as important. In fact, recent data confirmed the protective roles of antibodies in chlamydial infection in koalas and other animal studies [46, 47]. If antibodies do play a role in protection, then it will be via their neutralisation role. We therefore measured the *in vitro* neutralisation ability of plasma antibodies from animals immunised with the two adjuvants. We evaluated both their total neutralisation ability but we also determined which peptides within the MOMP protein the antibodies were directed against and which of these were the most important for the neutralisation effect. This produced very interesting and promising results for the neutralisation ability of plasma from vaccinated koalas.

Firstly, both adjuvants produced antibodies that were equally neutralising. This confirms that MOMP has B cell epitopes that can be neutralising, validating it as a good vaccine target. Interestingly, the adjuvants resulted in a different, but overlapping, set of vaccine-induced epitopes. Three peptides were recognised by both adjuvants (4, 28, and 41/42), but two additional epitopes (58/59 and 77) were solely recognised by Tri-Adj-immunised animals. The adsorption experiments nicely confirmed that several anti-epitope antibodies contributed to the *in vitro* neutralisation effect. Studies in the non-human primate model utilising native MOMP formulations had previously shown serovar- specific immune response either to homologous serovars [48] or cross-reacting to the closely related heterologous serovars [49]. Interestingly, in this study, the vaccine-induced epitopes recognised are all located in the conserved domains suggesting their role in cross-reactive recognition against diversified MOMP genotypes. Several vaccine studies have used the native form of MOMP, arguing that MOMP in its native should elicit a more robust immune response [48]. However, this study suggests that rMOMP is capable of generating neutralising epitopes in koalas. Nonetheless, 80% of the animals responded to epitope 77 in the variable region, but did not result in extra neutralising capacity.

In summary, both the adjuvants induce Th1-biased immune responses with neutralising antibodies. It is promising that the single dose Tri-Adj is able to produce a comparable immune response to the two or three-shot ISC up to 6 months time point. Tri-Adj has proven to be an effective adjuvant system for koala-*Chlamydia* vaccine design, and a practicable solution to eliminate multiple vaccination events. However, the longevity of the response elicited by each adjuvant in koalas remains to be determined. All of the surviving animals in our original study [16] that were immunised with the ISC adjuvant have high plasma antibody levels and memory CD4 cells 8 years after vaccination, while we don’t yet have similar data for the Tri-Adj. The identification of key epitopes (for the development of neutralising antibodies) enables future studies to focus on including these, or to develop specific assays to evaluate vaccine effectiveness.

## Supporting Information

S1 Fig Epitopes specificity of the two adjuvant cohorts as supporting documents. (PDF)
