# Supplementary figures and images for: Antibody and Cytokine Responses of Koalas (Phascolarctos cinereus) Vaccinated with Recombinant Chlamydial Major Outer Membrane Protein (MOMP) with Two Different Adjuvants
Source: PLoS One. 2016 May 24;11(5):e0156094. doi: 10.1371/journal.pone.0156094 (PMC4878773; doi:10.1371/journal.pone.0156094)

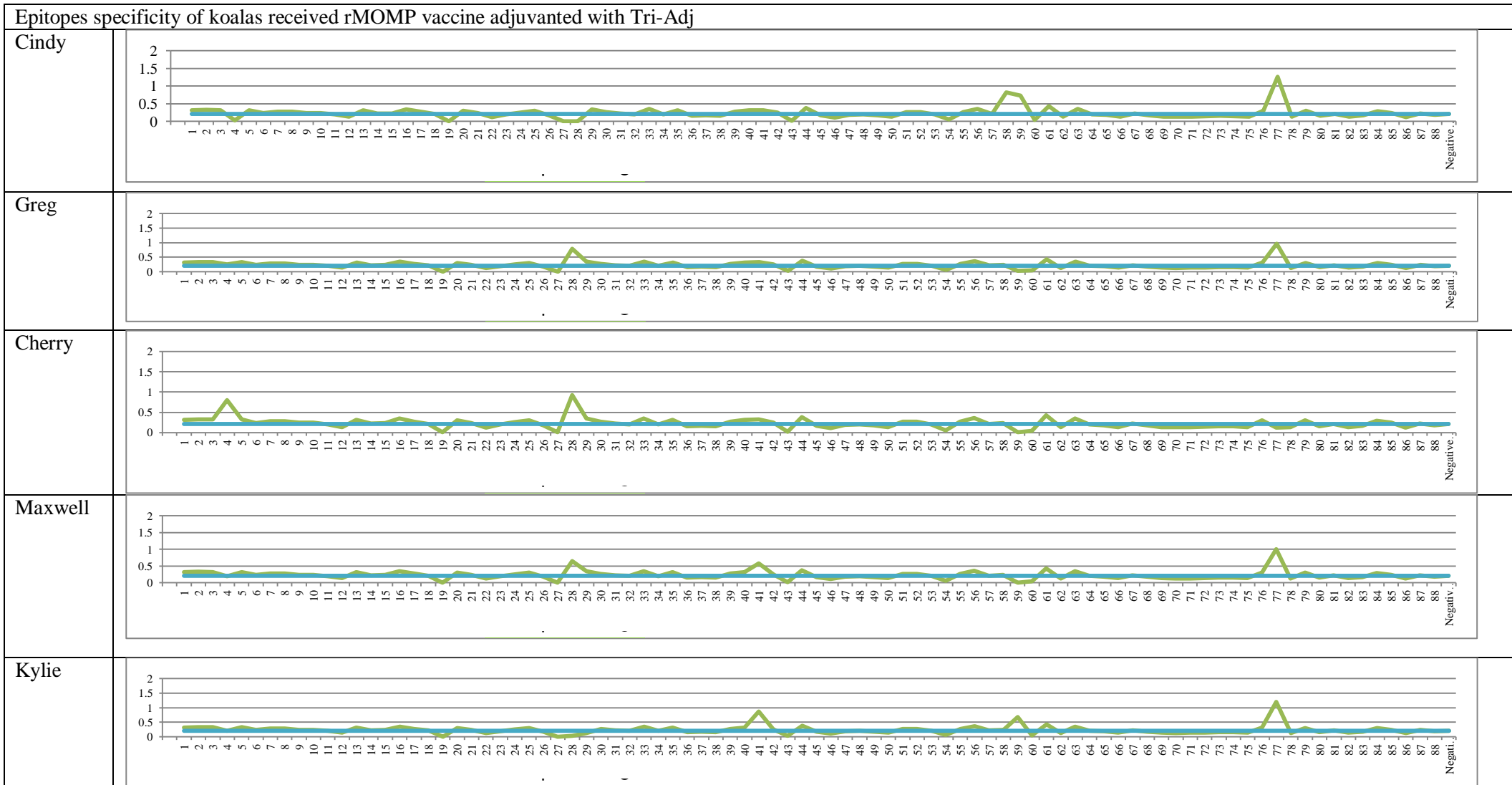

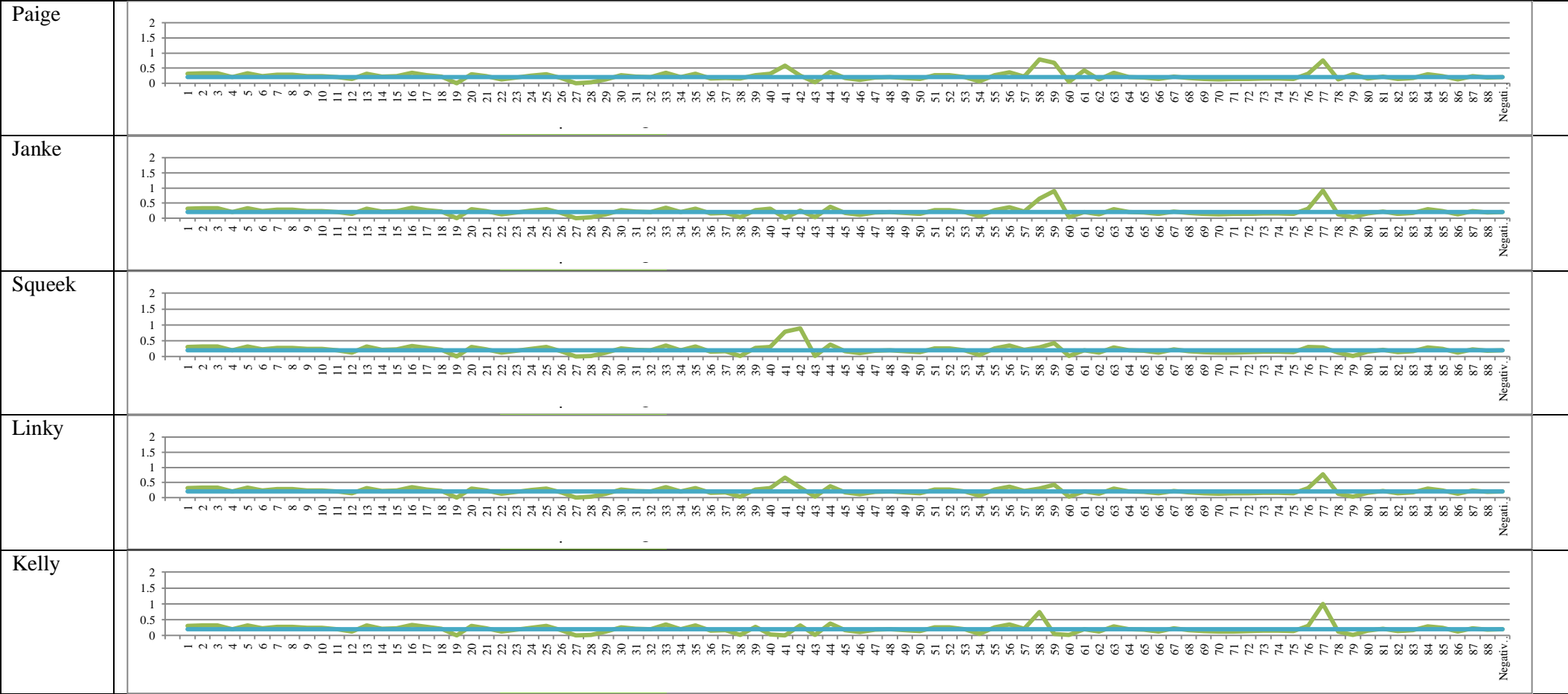

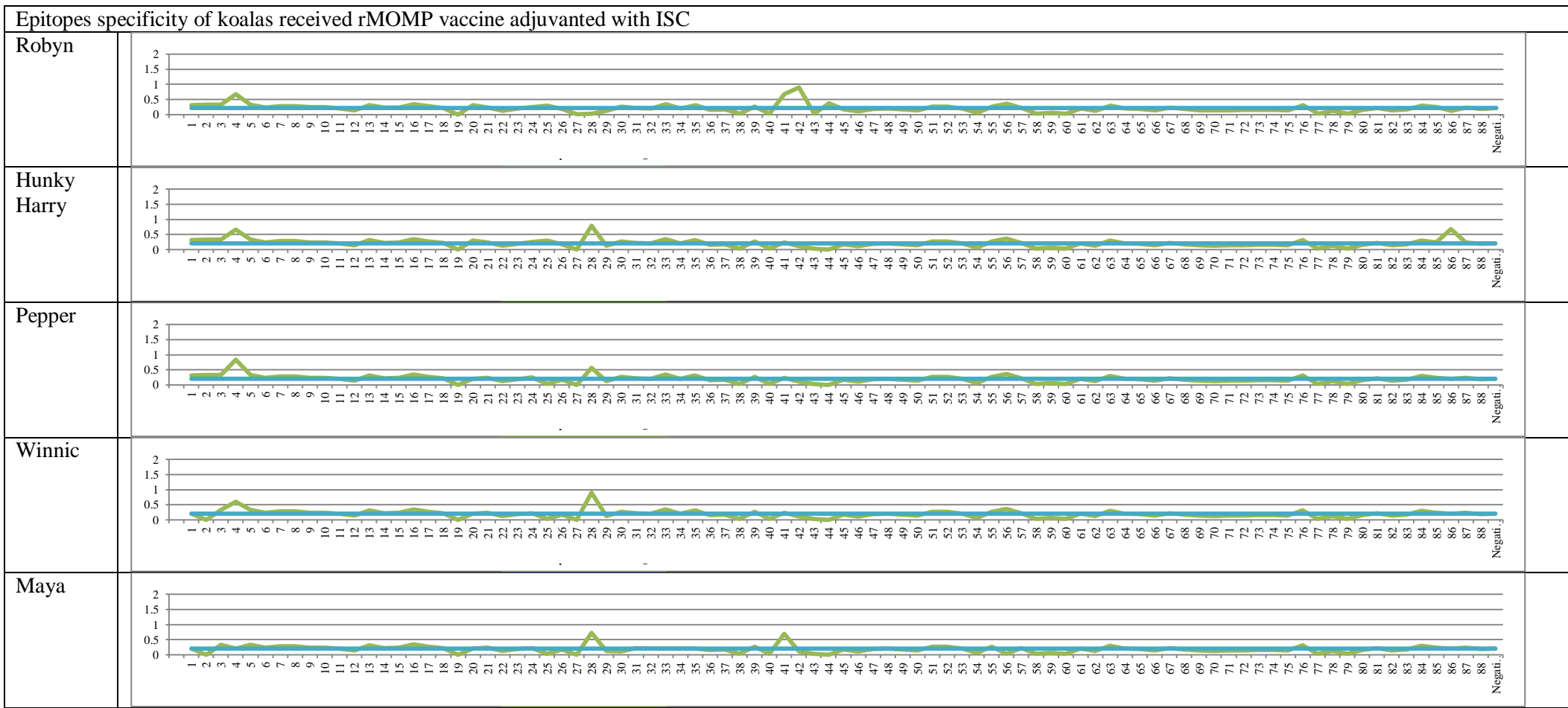

Supplement: S1 Fig — (PDF) [file pone.0156094.s001.pdf]
